# Radiocesium uptake in two fungal bed-cultivated edible mushroom species in forests in Fukushima, Japan

**DOI:** 10.1371/journal.pone.0342639

**Published:** 2026-08-03

**Authors:** Masaru Sakai, Mirai Watanabe, Masami Kanao Koshikawa, Seiji Furukawa, Seiichi Takechi, Kaoru Yoshida, Akiko Takahashi, Masanori Tamaoki, Masabumi Komatsu, Hajime Murai, Takashi Tsuji, Mai Takagi, Seiji Hayashi

**Affiliations:** 1 Fukushima Regional Collaborative Research Center, National Institute for Environmental Studies, Miharu, Fukushima, Japan; 2 Center for Watershed Sciences, University of California, Davis, California, United States of America; 3 Regional Environmental Conservation Division, National Institute for Environmental Studies, Tsukuba, Ibaraki, Japan; 4 Mushroom Promotion Center, Fukushima Prefectural Association of Forest, Forestry, and Revegetation, Koriyama, Fukushima, Japan; 5 Regional Environmental Co-creation Unit, Fukushima Institute for Research, Education and Innovation, Miharu, Fukushima, Japan; 6 Biodiversity Division, National Institute for Environmental Studies, Tsukuba, Ibaraki, Japan; 7 Department of Mushroom Science and Forest Microbiology, Forestry and Forest Products Research Institute, Tsukuba, Ibaraki, Japan; 8 Health and Environmental Risk Division, National Institute for Environmental Studies, Tsukuba, Ibaraki, Japan; Universiti Teknologi Malaysia, MALAYSIA

## Abstract

Mushrooms have long been valued as food resources, but past nuclear accidents disrupted production and shipment due to radiocesium contamination. After the Fukushima nuclear accident in 2011, limited knowledge has particularly hindered outdoor fungal bed cultivation. To address this, we conducted cultivation experiments with *Lyophyllum decastes* and *Lepista nuda* across 14 broad-leaved deciduous forest sites in Fukushima Prefecture. Air dose rates at 1-m height were 0.04–0.89 µSv/h. ^137^Cs concentrations (Bq/kg at 90% water content) in fruit bodies were 0.4–12 (mean 2.2) for *Ly. decastes* and 0.2–42 (mean 8.0) for *Le. nuda*. Both species remained below Japan’s food safety threshold of 100 Bq/kg, indicating that outdoor fungal bed cultivation is feasible for the species across broad areas. Uptake patterns differed: concentrations in *Ly. decastes* correlated with contamination in litter and multiple soil layers, whereas in *Le. nuda*, they correlated with upper soil and leaf mold contamination. These findings imply that clean soil fills for *Ly. decastes* and clean litter covers for *Le. nuda* may partially serve as mitigation strategies tailored to each species. Aggregated transfer factors (2.72 × 10^−5^ m^2^/kg for *Ly. decastes* and 5.72 × 10^−4^ m^2^/kg for *Le. nuda*) were one order of magnitude lower than those reported for wild mushrooms. Overall, this study provides new insights into reducing radiocesium assimilation by cultivated mushrooms and supports the revival of outdoor fungal bed cultivation in contaminated landscapes.

## Introduction

Mushrooms collected from forests and cultivated outdoors, serving as both seasonal delicacies and sources of rural livelihood, have long been central to food culture [1–3]. However, radiocesium contamination has posed a serious challenge to mushroom cultivation following nuclear accidents, such as those in Chernobyl in 1986 [4,5] and Fukushima in 2011 [6–8]. To prevent excessive radionuclide intake from such contamination events, the Codex Alimentarius established international guideline levels [9]. Specifically, the maximum limit for radiocesium (^134^Cs and ^137^Cs) in foods, including mushrooms, is set at 1,000 Bq/kg. Based on these guidelines, individual countries or regions have implemented their own thresholds, such as Japan’s stricter limit of 100 Bq/kg.

Fifteen years after the Fukushima Daiichi Nuclear Power Plant accident, the shipment of wild mushrooms is still restricted in 55 of 59 municipalities of Fukushima Prefecture due to ^137^Cs concentrations that exceed Japan’s limit [10]. In addition, 17 municipalities continue to restrict shipments of log-cultivated mushrooms grown outdoors [10]. To revive mushroom cultivation, science-based measures to produce less contaminated products are essential. A practical framework for addressing radiocesium contamination is to classify mushrooms into three groups—wild, log-cultivated, and fungal bed-cultivated—with the latter two further divided into outdoor and indoor systems.

Many species of edible wild mushrooms accumulate large amounts of radiocesium, although the transfer factors vary widely among species and ecological guilds (e.g., mycorrhizal versus saprophytic fungi) [6,7]. Soil improvements to mitigate radiocesium transfer are generally ineffective, as assimilation through extensive mycelial networks is too complex for localized interventions. Instead, scientific monitoring has identified species with lower transfer factors, which have been prioritized for collection, shipment, and consumption [6,7,11]. In fact, shipment restrictions for some of these low-accumulating species have been lifted in Japan since the Fukushima accident [12].

Among log-cultivated mushrooms, shiitake (*Lentinula edodes*) has been particularly affected, as Fukushima was one of its largest producers in Japan [13]. Radiocesium transfer from logs to mushrooms has been intensively studied [13–15], leading to government guidelines that have set a safe contamination threshold for logs (< 50 Bq/kg) and have outlined cultivation management practices [16]. Based on this scientific guidance, log-cultivated mushroom production has gradually resumed in both indoor and outdoor settings in Fukushima [17].

Among fungal bed-cultivated mushrooms, indoor systems have resumed in Fukushima under official guidance [16], which instructs farmers to prepare less contaminated fungal beds (< 200 Bq/kg), similar to log-cultivated mushrooms. Meanwhile, radiocesium assimilation in outdoor fungal bed systems, which are directly exposed to natural soils and forest litter, remains poorly understood, hindering cultivation and underscoring the need for further research. Studies conducted after the Fukushima and Chernobyl accidents reported initial radiocesium accumulation in organic horizons and subsequent transfer to subsurface soils [18]. Although radiocesium bioavailability is generally high in organic horizons [19], most radiocesium is now accumulating in subsurface soils in Fukushima [18]. Thus, outdoor fungal cultivation suffers persistently from nuclear accidents irrespective of the diverse radiocesium transfer patterns among species [6,7].

In this study, we examined radiocesium concentrations and environmental factors in two saprophytic mushroom species. Specifically, we investigated *Lyophyllum decastes* (wood/litter-decaying) and *Lepista nuda* (litter-decaying), using fungal bed systems at 14 broad-leaved deciduous forest sites in Fukushima, Japan. Both species are widely consumed in fried dishes and soups and had largely supported Fukushima’s mushroom industry before the accident. Wild *Ly. decastes* generally accumulates less radiocesium than other edible mushrooms, whereas *Le. nuda* tends to accumulate more [6]. This difference is thought to occur due to mycelial development patterns, as *Ly. decastes* mycelium extends mainly to buried woody materials, whereas *Le. nuda* mycelium extends to surrounding leaf molds [20,21]. Therefore, comparing assimilation patterns between these two species could identify species-specific strategies for reducing contamination. We evaluated the environmental factors influencing radiocesium uptake, identified the soil layer serving as the primary source of nutrients, and assessed measures for obtaining less contaminated products. Our findings provide key insights for reviving outdoor fungal bed cultivation in contaminated landscapes.

## Methods

### Study sites and environments

The 14 experimental sites were in the villages of Iitate, Katsurao, Kawauchi, and Hirata; the towns of Minamiaizu, Shimogo, and Hanawa; and the cities of Koriyama (two sites), Kitakata, Soma, Date, Motomiya, and Tamura (S1 Table). These are located in montane areas covered with broad-leaved deciduous forest dominated by Fagaceae species, typical of the Fukushima landscape. Field access did not require governmental permission because none of the sites were within the designated evacuation zone. Because the ultimate goal was to resume outdoor mushroom cultivation in inhabited zones, cultivation was not performed within the so-called ‘difficult-to-return’ zone, where ambient radiation levels remain relatively high. Approval was obtained from landowners at each site.

**Fig 1:**
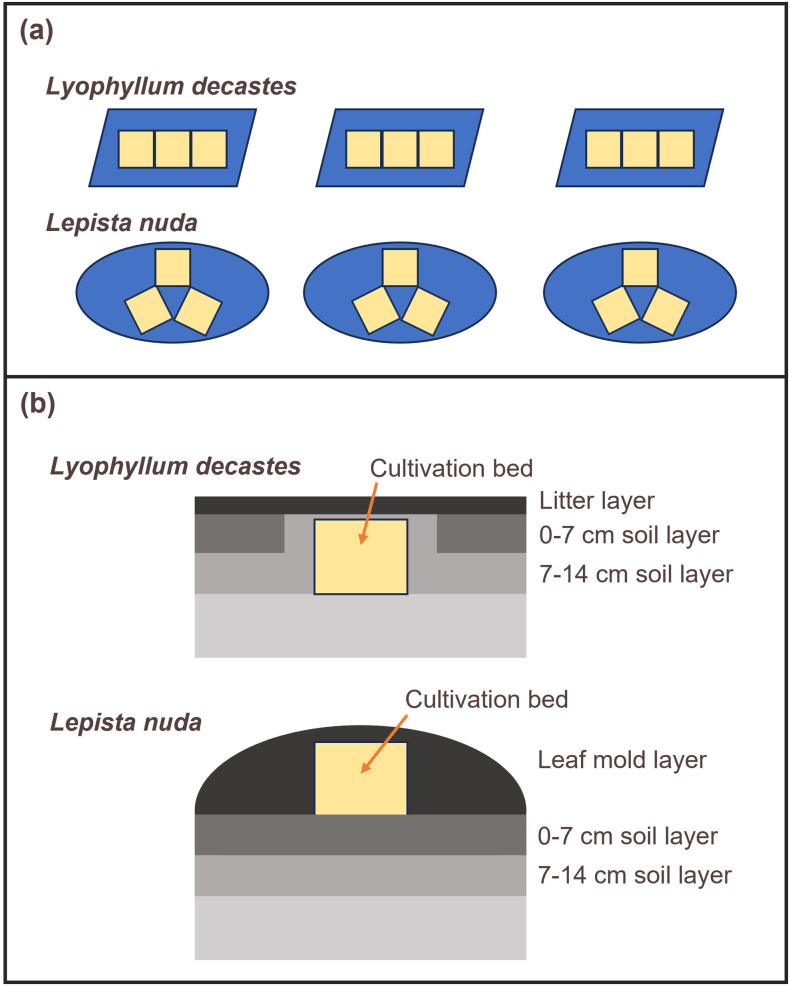
Layout and structure of cultivation beds (12 × 20 × 13 cm) for *Lyophyllum decastes* and *Lepista nuda* mushrooms in forests of Fukushima, Japan. (a) Top view of cultivation beds at the study site. (b) Cross-sectional view of cultivation beds and cover materials. Cultivation beds of *Ly. decastes* were placed on the top surface of a 14-cm-deep soil layer. The sides and top of these beds were fully covered with 7–14 cm of soil, which was further covered with litter. Cultivation beds of *Le. nuda* were placed on the topsoil and fully covered with leaf mold. The leaf mold cover was secured using wire nets and metal pegs to prevent wind disturbance. The distance between adjacent plots was 1 m across the six plots.

Cultivation beds of *Ly. decastes* and *Le. nuda* were planted at each site. The procedures for the cultivation experiments are summarized in Figs 1 and S1. Before bed placement, ambient gamma radiation dose rates at 1 m above each plot (three plots per species per site) were measured with a scintillation survey meter (TCS 172B; Hitachi Aloka Medical, Tokyo, Japan) equipped with a NaI(Tl) probe. These readings were primarily attributable to ^137^Cs and primordial radionuclides in soils, although values may have been slightly elevated by background radiation and instrument noise. Differences among sites were interpreted as reflecting ^137^Cs accumulation in the soil, because airborne radiation levels are a reliable proxy for ^137^Cs soil inventories in contaminated areas [22,23]. These measurements are also publicly available for broad areas of Fukushima Prefecture via a national airborne monitoring website [24] and can be obtained with portable dosimeters accessible to the public.

After measuring airborne radiation levels, the litter layer, 0–7-cm soil layer (i.e., fermentation and humus layers plus surface mineral soil), and 7–14-cm soil layer (i.e., subsurface mineral soil) were separately collected from three plots of *Ly. decastes* at each study site (Fig 1), because these strata contain most of the deposited ^137^Cs [18,25]. The total ^137^Cs soil inventory, as well as the inventories in the litter, 0–7-cm, and 7–14-cm layers (kBq/m^2^), were estimated from soil mass per unit area and ^137^Cs activity concentrations of the collected samples. The procedures for measuring ^137^Cs activity concentrations are described below. These inventory data were used as explanatory variables in statistical analyses for both species.

Meteorological conditions were monitored during cultivation experiments at each site (S1 Table). Air temperature, relative humidity, and sunlight intensity were recorded at 10-min intervals using sensors mounted on 1.7-m poles near the cultivation beds (temperature and humidity: HOBO U23-001; Onset, Bourne, MA, USA; sunlight: HOBO UA-002–64; Onset). Temperature and humidity loggers were housed in white instrument shelters, while sunlight sensors were installed at the top of the poles. Mean values of air temperature and relative humidity, together with daily duration of sunlight exceeding 2,000 lux during the cultivation periods of each plot, were used as explanatory variables in subsequent statistical analyses.

### Fungal bed cultivation

Three cuboid cultivation beds (12 × 20 × 13 cm) of *Ly. decastes* were placed in a hole (41 × 26 × 14 cm) created during soil sampling and fully covered with 7–14 cm of soil, reflecting the species’ preference for buried woody substrate (Figs 1 and S1). Then, the soil surface above each bed was covered with litter collected from the plots. In parallel, three cultivation beds of *Le. nuda* (12 × 20 × 13 cm) were placed on the mineral topsoil after removing the organic layers, and covered with leaf mold (litter mixed with fragmented organic material) collected from a 1-m-diameter circle in each plot, consistent with its litter-decaying habit (Figs 1 and S1). In total, 3 beds × 14 sites were established for each species.

The cultivation beds consisted of a fungus-inoculated 10:2 mixture of bark compost and bran. Because the ^137^Cs concentrations in the bark compost were low (~50 Bq/kg) and the bran was clean, ^137^Cs in the cultivated mushrooms was assumed to originate primarily from surrounding litter and soils. A preliminary study also indicated that ^137^Cs concentrations in the fruit bodies of both species were < 1 Bq/kg when cultivated on the fungal bed substrate alone (MW, unpublished data). These beds were standard commercial products identical to those sold to farmers in Fukushima before the 2011 nuclear accident.

Beds were planted between 23 July and 1 August 2024. *Ly. decastes* was harvested in the period 7–20 October 2024, and *Le. nuda* in the period 7–21 November 2024. Cultivation periods were 71–87 days for *Ly. decastes* and 102–118 days for *Le. nuda*, depending on growth. No water or fertilizers were supplied beyond natural rainfall. The total weight of mushrooms meeting shipping standards was recorded immediately after harvest.

### Radioactivity measurements

Litter and soil samples from *Ly. decastes* plots and leaf mold samples from *Le. nuda* plots were air-dried for at least 1 month. Litter and leaf mold were fragmented with scissors and sieved through 9.5-mm mesh, while soils were sieved through 2-mm mesh. Each sample was packed into a 100-mL plastic container. Dry weight was determined from the weight loss of subsamples dried at 105°C for ≥ 24 h, while bulk samples were stored at 25°C for other chemical analyses.

Fresh mushrooms were gently washed with tap water to remove soil particles and organic debris, wiped, and dried at 60°C for ≥ 2 days. Drying at 60°C was chosen to minimize loss of volatile organic matter compared to drying at 105°C. Dried samples were finely ground with an electric mill and packed into 100-mL containers. Dry weights and sample densities were recorded prior to radioactivity measurements.

^137^Cs activity concentrations in litter, leaf mold, soil, and mushroom samples were determined via gamma-ray spectroscopy. Activity concentrations of ^134^Cs and ^137^Cs were determined from their gamma-ray peaks at 604.66 and 661.6 keV, respectively, using a coaxial high-purity germanium detector (GC2020; Canberra Japan, Tokyo, Japan) operated with Spectrum Explorer software (Canberra Japan, Tokyo, Japan). Mean relative counting uncertainty (1σ), calculated as error counts divided by net peak counts, was approximately 5% for litter, leaf mold and soil samples and 8% for mushrooms. Counting times ranged from 10,000–200,000 s. All reported activities were decay corrected to the sampling date. Mushroom concentrations were expressed on fresh weight bases, with fresh weight calculated assuming 90% water content [6,26].

Gamma-ray spectra were analyzed using an efficiency calibration file prepared from measurements of the standard volume radioactivity source MX033U8PP (Japan Radioisotope Association, Tokyo, Japan), consisting of an alumina matrix in the same 100-mL geometry as the sample containers. Sample density and height were also considered when calculating activity concentrations. Coincidence summing correction for ^134^Cs was applied. Peak detection was based on the Cooper method (detection limit factor = 3.0). It was defined as the activity expected to produce a measured result exceeding the 3σ detection criterion with a probability of 50%. Routine quality assurance included monthly background measurements, routine performance checks using standard reference sources, and annual detector inspection.

Because ^134^Cs was below the detection limit in all mushroom samples, total radiocesium (^134^Cs + ^137^Cs) activity concentrations were estimated from measured ^137^Cs activity concentrations [27]. We assumed that all measured ^137^Cs originated from the Fukushima accident and that the initial emissions of ^137^Cs and ^134^Cs were equal. Measured ^137^Cs activity concentrations in mushrooms were decay-corrected to 11 March 2011 using the ^137^Cs half-life (30.16 years) and used to estimate the corresponding ^134^Cs activity concentrations on that date. The estimated ^134^Cs activity concentrations were then decay-corrected to the sampling date using the ^134^Cs half-life (2.06 years) and added to the measured ^137^Cs activity concentrations to obtain total radiocesium (^134^Cs + ^137^Cs) activity concentrations. These values are presented only in S1 Table for reference since Japan’s food safety threshold of 100 Bq/kg includes both ^134^Cs and ^137^Cs activity concentrations. Meanwhile, all subsequent statistical analyses were performed using measured ^137^Cs activity concentrations.

### Statistical analyses

First, we constructed a linear mixed model (LMM) to examine the relationship between total ^137^Cs soil inventory (kBq/m^2^) and airborne radiation levels at 1-m height (µSv/h). We initially compared the Akaike information criterion (AIC) among four models (all combinations of raw or log_10_-tranformed data, and the LMM or a simple linear model [LM]). Ultimately, we chose the LMM with log_10_-tranformed data, as it yielded the lowest AIC (a delta of > 20 with the second-best model). Thus, the response variable was log_10_-transformed total ^137^Cs soil inventory, the explanatory variable was log_10_-transformed radiation level, and study site identifiers were included as random intercepts to account for site variability. A Gaussian error structure was assumed. The resulting regression was used to estimate an upper threshold of airborne radiation at which ^137^Cs concentrations in mushrooms could exceed the Japanese food safety limit of 100 Bq/kg, contingent on statistically significant relationships between mushroom ^137^Cs concentrations and soil ^137^Cs inventories.

Second, we constructed LMs for each species to evaluate factors that influenced ^137^Cs concentrations in mushrooms. To account for potential site-specific variability, we initially constructed LMMs with ‘site’ as a random effect. These were compared against simpler LMs using the AIC. For both mushroom species, the LMs yielded substantially lower AIC values (a delta of > 20), indicating that the fixed environmental predictors were sufficient to capture the variance and that the inclusion of random effects led to unnecessary model complexity. Explanatory variables included log_10_-transformed total ^137^Cs soil inventory (kBq/m^2^), mean relative humidity (%), total mushroom yield (g), cultivation period (days), mean daily duration of sunlight > 2,000 lux (h/day), and mean air temperature (°C). The response variable was the log_10_-transformed ^137^Cs activity concentration in mushrooms. Soil inventories estimated in *Ly. decastes* plots were assigned to adjacent *Le. nuda* plots because disturbances resulting from soil sampling made it difficult to evaluate ^137^Cs transfer from soil to *Le. nuda* mushrooms. Explanatory variables were standardized (mean = 0, standard deviation [SD] = 0.5) prior to analysis [28] to allow direct comparison of coefficients.

Model diagnostics were performed using a simulation-based approach to create standardized quantile residuals [29]. This allowed a robust assessment of model assumptions, including normality, heteroscedasticity, and potential site-specific biases. The residual plots confirmed that the LMs met all statistical assumptions, with no significant systematic deviations detected across different sites or predictor ranges (*P* > 0.05). Multicollinearity was negligible (|*r*| < 0.7, variance inflation factor < 3). Final model selection was conducted via an automated dredge function [30] to identify the most parsimonious set of predictors based on AIC weights.

Third, six additional LMMs were constructed following the AIC-based assessments between LMMs and LMs, each with a single explanatory variable (log_10_-transformed ^137^Cs inventories or activity concentrations in litter, 0–7-cm soil, or 7–14-cm soil) to identify the major ^137^Cs sources of the mushrooms. For *Le. nuda*, an LMM with log_10_-transformed ^137^Cs activity concentration in leaf mold was also tested, given its likely role as a primary source. The response variable was the log_10_-transformed ^137^Cs activity concentration in mushrooms. Site identifiers were included as random intercepts. All LMMs were fitted using the lmer function in the lme4 package [31]; explanatory variables were standardized with the arm package [32]; model diagnostics were performed using the DHARMa package [29]; model selections were performed using the MuMIn package [30] in R 4.5.1 [33]. Statistical significance was set at *P* < 0.05 using the lmerTest package [34].

Finally, aggregated transfer factors for each species were calculated based on Equation (1):


$$\begin{array}{c} Aggregated\;transfer\;factor={(}^{\text{1}\text{3}\text{7}}Cs\;conc.\;in\;mushroom)\;/\;(total\text{1}\text{3}\text{7}Cs\;soil\;inventory) \end{array}$$ (1)

Then, the geometric means ± geometric SD of the aggregated transfer factors (m^2^/kg) were calculated, and compared to values reported in previous studies.

## Results

**Fig 2:**
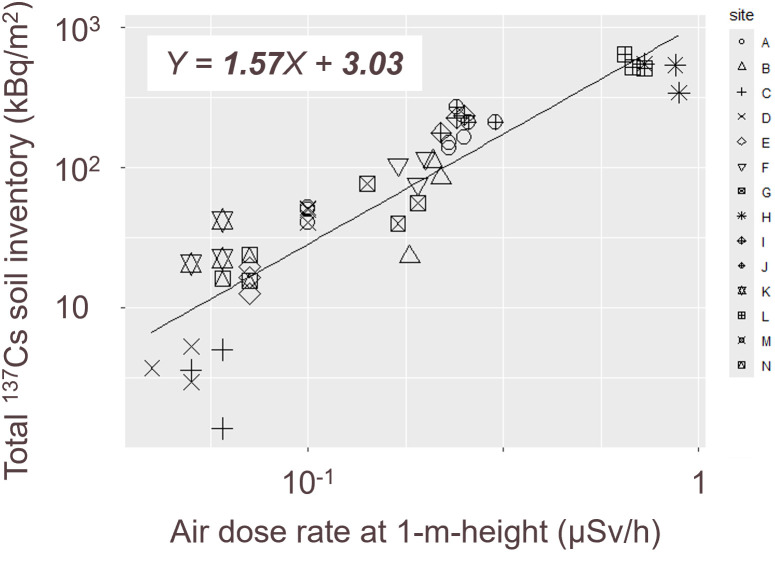
Relationship between the total ^137^Cs soil inventory and the air dose rate at 1-m height. Symbols represent individual study sites, with different shapes assigned to each site. The slope and intercept values were statistically significant (*P* < 0.05).


Air dose rates at 1-m height were 0.04–0.89 µSv/h, and total ^137^Cs soil inventories were 1.39–640 kBq/m^2^ across the study sites. The LMM indicated a significant positive relationship between them (Fig 2), expressed as:

$$\begin{array}{c} {log}_{\text{1}\text{0}}\left(total\text{1}\text{3}\text{7}Cs\;soil\;inventory\right)=\text{1}.\text{5}\text{7}\;\times \;{log}_{\text{1}\text{0}}(air\;dose\;rate)-\text{3}.\text{0}\text{3} \end{array}$$ (2)

^137^Cs concentrations in mushrooms were in the range 0.4–12 Bq/kg (mean 2.2) in *Ly. decastes* and 0.2–42 Bq/kg (mean 8.0) in *Le. nuda* (90% water content basis). No samples exceeded the Japanese food safety limit of 100 Bq/kg.

**Fig 3:**
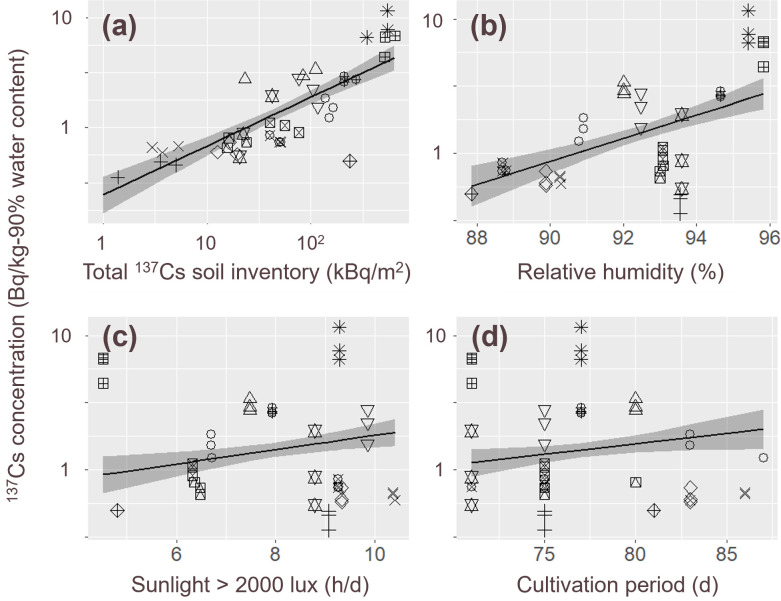
Relationships between the ^137^Cs activity concentration in *Lyophyllum decastes* mushrooms and environmental variables. (a) Total ^137^Cs soil inventory, (b) mean relative humidity, (c) mean duration of sunlight > 2,000 lux, and (d) cultivation period. Regression lines represent statistically significant relationships identified by the best linear model, with other explanatory variables fixed at their mean values. Shaded bands indicate 95% confidence intervals. Different symbols denote individual study sites, as indicated in Fig 2.

For *Ly. decastes*, the best LM indicated that total ^137^Cs soil inventory, relative humidity, mean daily duration of sunlight > 2,000 lux, and cultivation period were significantly positively correlated with mushroom ^137^Cs concentrations (Table 1, Fig 3). Based on Equation (2), concentrations were estimated to exceed 100 Bq/kg at an air dose rate of 65.71 µSv/h, with other explanatory variables fixed at mean values.

**Table 1:** Results of the best linear model testing the effects of total ^137^Cs inventory in soil, mean relative humidity, mean duration of sunlight > 2,000 lux, and cultivation period on ^137^Cs activity concentrations in *Lyophyllum decastes* mushrooms. The estimated values were standardized to enable comparison among the explanatory variables. Statistically significant values are shown in bold.

	Estimate	SE	*t*	*P*
Total ^137^Cs inventory in soil	0.62	0.06	9.75	**0.00**
Relative humidity	0.38	0.07	5.69	**0.00**
Sunlight > 2000 lux	0.18	0.06	3.00	**0.00**
Cultivation period	0.14	0.06	2.28	**0.03**

**Fig 4:**
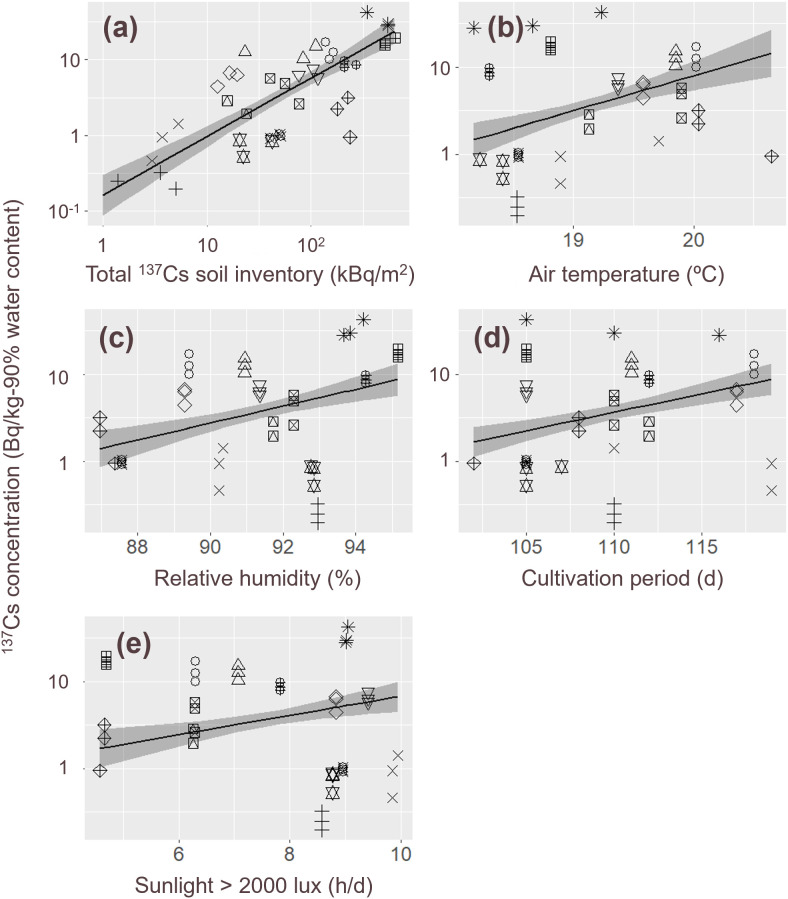
Relationships between the ^137^Cs activity concentration in *Lepista nuda* mushrooms and environmental variables. (a) Total ^137^Cs soil inventory in soil, (b) mean air temperature, (c) mean relative humidity, (d) cultivation period, and (e) mean duration of sunlight > 2,000 lux. Regression lines represent statistically significant relationships identified by the best linear model, with other explanatory variables fixed at their mean values. Shaded bands indicate 95% confidence intervals. Different symbols denote individual study sites, as indicated in Fig 2.

For *Le. nuda*, the best LM indicated that total ^137^Cs soil inventory, mean air temperature, relative humidity, cultivation period, and mean daily duration of sunlight > 2,000 lux were significantly positively correlated with mushroom ^137^Cs concentrations (Table 2, Fig 4). Exceedance was estimated at 2.34 µSv/h under mean conditions.

**Table 2:** Results of the best linear model testing the effects of total ^137^Cs inventory in soil, mean air temperature, mean relative humidity, cultivation period, and mean duration of sunlight > 2,000 lux on ^137^Cs activity concentrations in *Lepista nuda* mushrooms. The estimated values were standardized to enable comparison among the explanatory variables. Statistically significant values are shown in bold.

	Estimate	SE	*t*	*P*
Total ^137^Cs inventory in soil	1.09	0.10	10.89	**0.00**
Air temperature	0.54	0.12	4.65	**0.00**
Relative humidity	0.47	0.10	4.51	**0.00**
Cultivation period	0.41	0.09	4.64	**0.00**
Sunlight > 2000 lux	0.38	0.11	3.44	**0.00**

**Fig 5:**
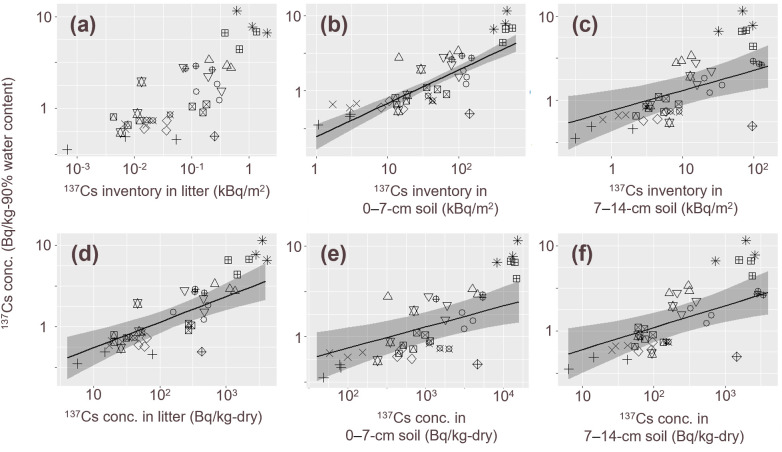
Relationships between the ^137^Cs activity concentration in *Lyophyllum decastes* mushrooms and the ^137^Cs inventory and concentration in litter and soils. ^137^Cs inventories in (a) litter, (b) 0–7-cm soil, and (c) 7–14-cm soil. ^137^Cs activity concentrations in (d) litter, (e) 0–7-cm soil, and (f) 7–14-cm soil. Regression lines represent statistically significant relationships identified by linear mixed models. Shaded bands indicate 95% confidence intervals. Different symbols denote individual study sites, as indicated in Fig 2.

**Fig 6:**
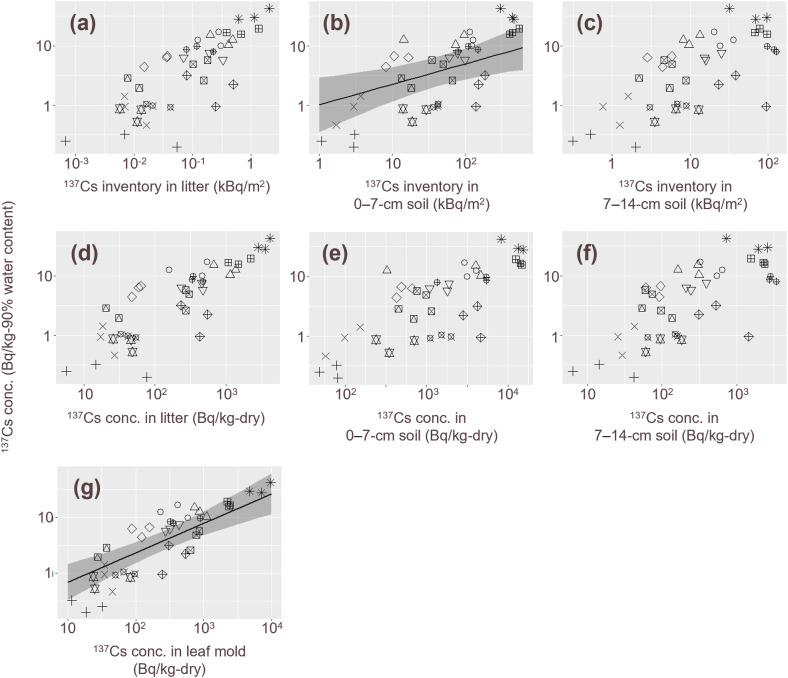
Relationships between the ^137^Cs activity concentrations in *Lepista nuda* mushrooms and ^137^Cs inventories and activity concentrations in litter, soil, and leaf mold. ^137^Cs inventories in (a) litter, (b) 0–7-cm soil, and (c) 7–14-cm soil. ^137^Cs activity concentrations in (d) litter, (e) 0–7-cm soil, (f) 7–14-cm soil, and (g) leaf mold. Regression lines represent statistically significant relationships identified by linear mixed models. Shaded bands indicate 95% confidence intervals. Different symbols denote individual study sites, as indicated in Fig 2.

Species-specific source relationships were evident (Figs 5, 6). For *Ly. decastes*, ^137^Cs inventories in 0–7-cm and 7–14-cm soils, and ^137^Cs activity concentrations in litter and both soil layers were significantly positively correlated with mushroom ^137^Cs concentrations (Table 3, Fig 5). In *Le. nuda*, the ^137^Cs inventory in 0–7-cm soil and the activity concentration in leaf mold were significantly positively correlated (Table 3, Fig 6).

**Table 3:** Results of 13 linear mixed models testing the effects of ^137^Cs inventory and concentration in litter, 0–7-cm soil, and 7–14-cm soil on ^137^Cs activity concentrations in *Lyophyllum decastes* and *Lepista nuda* mushrooms, and the effect of ^137^Cs concentration in leaf mold on ^137^Cs concentrations in *Le. nuda* mushrooms. Statistically significant values are shown in bold.

	Estimate	SE	*t*	*P*
*Lyophyllum decastes*				
^137^Cs inventory in litter	0.10	0.06	1.74	0.09
^137^Cs inventory in 0–7-cm-depth soil	0.31	0.08	3.96	**0.00**
^137^Cs inventory in 7–14-cm-depth soil	0.24	0.08	3.03	**0.00**
^137^Cs concentration in litter	0.31	0.07	4.60	**0.00**
^137^Cs concentration in 0–7-cm-depth soil	0.23	0.07	3.28	**0.00**
^137^Cs concentration in 7–14-cm-depth soil	0.26	0.08	3.28	**0.00**
*Lepista nuda*				
^137^Cs inventory in litter	0.00	0.07	0.00	1.00
^137^Cs inventory in 0–7-cm-depth soil	0.34	0.12	2.93	**0.01**
^137^Cs inventory in 7–14-cm-depth soil	–0.03	0.11	–0.31	0.76
^137^Cs concentration in litter	0.09	0.11	0.79	0.44
^137^Cs concentration in 0–7-cm-depth soil	0.19	0.10	1.89	0.07
^137^Cs concentration in 7–14-cm-depth soil	–0.05	0.11	–0.50	0.62
^137^Cs concentration in leaf mold	0.53	0.10	5.41	**0.00**

The aggregated transfer factors were 2.72 × 10^−5^ ± 1.17 m^2^/kg for *Ly. decastes* and 5.72 × 10^−4^ ± 1.19 m^2^/kg for *Le. nuda*.

## Discussion

This study confirmed that ^137^Cs activity concentrations in *Ly. decastes* and *Le. nuda* mushrooms, cultivated in broad-leaved deciduous forests where ambient dose rates ranged from 0.04 to 0.89 µSv/h at 1-m height, were well below the Japanese food safety limit of 100 Bq/kg. These findings imply that both species generally exhibit low ^137^Cs accumulation when cultivated in most areas outside the evacuation zone, where current ambient dose rate are below 1 µSv/h [24]. Moreover, these results provide practical insights into mitigation measures for ^137^Cs assimilation in cultivated mushrooms.

For *Ly. decastes*, ^137^Cs concentrations were consistently low (0.4–12 Bq/kg) but had the strongest positive correlation with the total ^137^Cs soil inventory, indicating slight assimilation from soil proportional to contamination levels. Because *Ly. decastes* forms clustered mushrooms [35] and fruit bodies emerge exclusively from the top surfaces of cultivation beds, ^137^Cs in surrounding soils critically influences mushroom ^137^Cs concentrations [36]. Similar positive relationships have been reported in certain wild vegetable species [23,37], underscoring that cultivation outside high-radiation areas is a prerequisite for producing crops with low enough ^137^Cs concentrations. Relative humidity also exhibited a positive correlation with mushroom radiocesium concentrations. Because soil moisture strongly influences ^137^Cs bioavailability [38] and fungal uptake [39], higher humidity may indirectly increase assimilation in *Ly. decastes*. Sunlight and cultivation period were also positively correlated with mushroom ^137^Cs concentrations, implying that an increase in water demand due to intensified sunlight [40] and longer exposure to contaminated soils can elevate mushroom ^137^Cs concentrations [36], although these assumptions need to be verified by additional experiments.

For *Ly. decastes*, ^137^Cs inventories in 0–7-cm and 7–14-cm soils, as well as ^137^Cs activity concentrations in litter and both soil layers, were positively correlated with mushroom ^137^Cs concentrations. These results imply assimilation from both litter and multiple soil horizons. Our data indicate that ^137^Cs was stored primarily in 0–7-cm soil (mean 77% of total), followed by 7–14-cm soil (mean 23%), and litter (mean 0.23%), consistent with previous studies [18,27]. In contrast, ^137^Cs bioavailability is generally higher in organic layers and decreases with depth [41,42]. This balance likely explains the observed transfers to *Ly. decastes* from multiple layers. Because the cultivation beds were placed in holes that were filled with a 7–14-cm soil layer, and the soil surface above each bed was covered with litter (Fig 1), the cultivation beds were in direct contact with the 7–14-cm soil. Meanwhile, the surrounding forest floor retained the natural soil profile, consisting of a litter layer overlying 0–7-cm and 7–14-cm soil layers. Thus, although the cultivation beds contacted the 7–14-cm soil layer directly, they could also be indirectly influenced by ^137^Cs present in the adjacent litter, 0–7-cm soil, and 7–14-cm soil. Consequently, *Ly. decastes* could assimilate ^137^Cs from all layers. Practically, filling cultivation bed holes with less contaminated soil, as done here, appears to be an effective mitigation measure because surrounding soils are the primary ^137^Cs sources for *Ly. decastes* mushrooms [36]. This should be verified through manipulation experiments using soil fills with various levels of contamination.

For *Le. nuda*, mushroom ^137^Cs concentrations (0.2–43 Bq/kg) remained below national safety limits for shipment and consumption. As with *Ly. decastes*, the total ^137^Cs soil inventory was positively correlated with mushroom contamination, implying that cultivation at less contaminated sites is a promising strategy. Relative humidity, cultivation period, and sunlight were similarly positively correlated with mushroom ^137^Cs concentrations in *Ly. decastes*. Although manipulation experiments are required to test the causality of these relationships, it appears that common responses of ^137^Cs in mushrooms to such environmental conditions can occur in these litter-decaying and wood/litter-decaying fungi. ^137^Cs concentrations in *Le. nuda* additionally exhibited a positive relationship with air temperature. While the mean temperature was 18.2–20.7°C in our study, the optimal temperature for mycelial growth in this species is estimated to be 24°C [43]. Because mycelial extension to contaminated leaf molds can result in increased ^137^Cs assimilation, a positive relationship might occur in this temperature range.

When focusing on direct relationships, ^137^Cs concentrations in *Le. nuda* mushrooms were higher at sites with greater ^137^Cs in leaf mold. Because *Le. nuda* is a litter-decaying fungus that extends mycelia into surrounding litter and produces fruiting bodies from it [20,21], these results confirm that assimilation occurs primarily from the leaf mold covering cultivation beds. The ^137^Cs inventory in 0–7-cm soil was also significantly positively related to mushroom ^137^Cs concentrations. Thus, the relatively high ^137^Cs accumulation in this soil layer influenced levels in *Le. nuda* mushrooms, even though their primary ^137^Cs source was leaf mold. Although ^137^Cs concentrations in mushrooms were low at our study sites, maintaining clean leaf mold cover during cultivation may be an effective mitigation measure. This should be tested through manipulation experiments using leaf mold covers with various contamination levels, given that our results also showed a positive relationship between ^137^Cs accumulation in mushrooms and the 0–7-cm soil layer.

Our results confirm that *Ly. decastes* and *Le. nuda* mushrooms contained ^137^Cs concentrations low enough to meet the national safety limit for shipment and consumption, and were broadly obtainable throughout broad-leaved deciduous forests with < 1 µSv/h. The geometric mean of the aggregated transfer factor was one order of magnitude lower in this study for *Ly. decastes* (2.7 × 10^−5^ m^2^/kg) than for previously reported wild *Ly. decastes* mushrooms (1.0 × 10^−4^ m^2^/kg in 2011–2017 [6] and 4.0 × 10^−4^ m^2^/kg in 2016–2020 [39]). For *Le. nuda*, the value was also one order of magnitude lower (5.7 × 10^−4^ m^2^/kg) than those of wild *Le. nuda* mushrooms (2.4 × 10^−3^ m^2^/kg in 2011–2017 [6] and 2.8 × 10^−3^ m^2^/kg in 2016–2020 [39]). These results imply that both temporal changes and cultivation environments (wild versus managed beds) are critical factors determining ^137^Cs contamination in these mushroom species, and highlight the potential for outdoor cultivation to provide less contaminated products compared to wild mushroom collection.

Our findings further imply that filling cultivation bed holes with clean soil for *Ly. decastes* and preparing clean leaf mold cover for *Le. nuda* may reduce contamination in cultivated mushrooms. Nonetheless, our study had limitations that warrant future research. First, the thresholds at which *Ly. decastes* (65.71 µSv/h) and *Le. nuda* (2.34 µSv/h) mushrooms would exceed 100 Bq/kg were extrapolated based on our low-dose-rate sites (0.04–0.89 µSv/h) and, thus, these estimations remain uncertain. For example, highly contaminated sites may provide ^137^Cs to mushrooms not only from litter and soil but also from canopy layers [19,44], implying that ^137^Cs concentrations in mushrooms might reach 100 Bq/kg even at lower-dose-rate sites. Therefore, additional experiments at more contaminated sites are necessary to confirm the actual thresholds. Second, the experiments were confined to broad-leaved deciduous forests, although different assimilation patterns may occur in other forest types such as evergreen coniferous forests [19,44]. Broader assessments across multiple forest types are needed. For example, since *Le. nuda* accumulates potassium from mycelial networks extending into leaf mold [45], the difference in potassium content of litter among forest types may influence ^137^Cs accumulation in this species. Third, soil adhering to mushrooms, which was removed in this study, can substantially increase measured ^137^Cs concentrations. This should be considered in future work. Fourth, we did not quantify the relative contributions of surrounding natural materials (litter and soil) and fungal bed substrates to mushroom contamination. To establish robust guidelines for bed preparation and cultivation practices, it is important to do so. Finally, additional plot-scale replications would improve the robustness.

## Conclusion

To date, limited knowledge regarding the ^137^Cs transfers to edible mushrooms cultivated outdoors has hindered the resumption of outdoor mushroom cultivation in contaminated areas. This study provides the first evidence that *Ly. decastes* and *Le. nuda* can be successfully cultivated across a broad range of contamination levels in broad-leaved deciduous forests. Science-based approaches of this kind are crucial to revive local mushroom cultivation in polluted regions. However, several limitations remain in this study. First, the contamination level of the soils below the cultivation beds of *Le. nuda* was not measured, which hinders the estimation of the actual ^137^Cs transfer from soils to mushrooms. Therefore, an additional experiment that controls soil contamination levels for *Le. nuda* cultivation is necessary. Second, our estimation of ^134^Cs concentrations in mushrooms depends on the assumption that ^134^Cs and ^137^Cs were emitted in equivalent proportions from the nuclear power plant. While the ^134^Cs concentration in mushrooms was below the detection limits, this estimation still includes uncertainty regarding ^134^Cs concentrations. Third, the proposed methods to reduce ^137^Cs transfer to mushrooms (filling with uncontaminated soil for *Ly. decastes* and covering with uncontaminated leaf mold for *Le. nuda*) have yet to be explicitly examined. These proposals are still preliminary and thus should be experimentally validated in future work. Resolving these limitations for the two species and expanding research to other edible mushroom species are essential to advance contamination-management strategies in outdoor fungal bed cultivation.

## Supporting information

S1 Table Sakai et al data. Radiocesium uptake in two fungal bed-cultivated edible mushroom species in forests in Fukushima, Japan. The table includes all data described in this paper (i.e., the mushroom ^137^Cs activity concentrations in each species, and the environmental variables).
(XLSX)

S1 Fig Cultivation bed placements, cover materials on cultivation beds, and fruit bodies of *Lyophyllum decastes* (left panel) and *Lepista nuda* (right panel). Picture Credit: Dr. Mirai Watanabe.
(PDF)
